# Hospital Readmission in Stroke Survivors in Social Vulnerability: Predictive Modeling with Machine Learning from the Perspective of the Chronic Conditions Care Model

**DOI:** 10.3390/ijerph22111705

**Published:** 2025-11-11

**Authors:** Erisonval Saraiva da Silva, Thereza Maria Magalhães Moreira, Ana Célia Caetano de Souza, Ana Maria Ribeiro dos Santos, Ana Roberta Vilarouca da Silva, Lariza Martins Falcão, Livia Carvalho Pereira, Jardeliny Corrêa da Penha, Manoel Borges da Silva Junior, Francisco Lucas de Lima Fontes, Isaias Wilmer Dueñas Sayaverde, Maria del Pilar Serrano Gallardo, José Wicto Pereira Borges

**Affiliations:** 1 Postgraduate Program in Nursing, Federal University of Piauí (UFPI), Teresina 64.049-550, PI, Brazil; ana.mrsantos@gmail.com (A.M.R.d.S.); robertavilarouca@yahoo.com.br (A.R.V.d.S.); liviacpereira4@gmail.com (L.C.P.); lucasfontesenf@ufpi.edu.br (F.L.d.L.F.); wictoborges@ufpi.edu.br (J.W.P.B.); 2 Postgraduate Program in Clinical Care in Nursing, State University of Ceará (UECE), Fortaleza 60.714-903, CE, Brazil; thereza.moreira@uece.br; 3 Walter Cantídio University Hospital, Federal University of Ceará (UFC), Fortaleza 60.020-181, CE, Brazil; anaceliacs.doc@gmail.com; 4 Nursing Department, Federal University of Piauí (UFPI), Teresina 64.049-550, PI, Brazil; lariza@ufpi.edu.br; 5 Postgraduate Program in Health and Community, Federal University of Piauí (UFPI), Teresina 64.000-020, PI, Brazil; jardeliny.penha@ufpi.edu.br (J.C.d.P.); manoelborges@ufpi.edu.br (M.B.d.S.J.); 6 School of Nursing, National Autonomous University of Chota (UNACH), Chota-Cajamarca 06421, Peru; iduenias@unach.edu.pe; 7 Nursing Department, Faculty of Medicine, Universidad Autónoma de Madrid, 28040 Madrid, Spain; pilar.serrano@uam.es

**Keywords:** stroke, decision trees, logistic models, chronic disease, healthcare models, social vulnerability, patient readmission

## Abstract

Hospital readmission among stroke survivors is frequent, especially in contexts of social vulnerability, compromising recovery and overburdening health services. This study aimed to develop a predictive model of hospital readmission among socially vulnerable stroke survivors, based on the Chronic Conditions Care Model (CCCM). Machine learning algorithms were applied, specifically decision tree and logistic regression, with data split into training (70% and 80%) and testing (30% and 20%) sets. Analyses were conducted using Python, with accuracy evaluated through ROC curves, AUC, and the confusion matrix in Analyse-it^®^, adopting a 5% significance level. The decision tree with an 80/20 partition achieved an accuracy of 92.45%. The variables most associated with readmission were falls, time since the first stroke, presence of a caregiver, and difficulty sleeping. In logistic regression, falls increased the risk by 235%, ischemic stroke by 155%, complications by 153.53%, COVID-19 by 132%, and time since stroke by 11.5% per year. The model proved to be feasible and robust, with the decision tree standing out, highlighting its potential to support preventive strategies and enhance care management.

## 1. Introduction

In Latin America, predictive models have been developed to estimate population health outcomes [1]. In countries such as Colombia [2] and Mexico [3], recent studies have applied Machine Learning (ML) techniques to predict outcomes in chronic diseases, including diabetes, hypertension, and stroke, demonstrating their potential to support clinical decision-making in contexts marked by inequality and fragmented health systems. In Brazil, the SABE Study employed ML algorithms to predict the five-year mortality risk among older adults [4].

In the context of non-communicable diseases (NCDs), predictive models show promise for improving health service organization. They provide prognostic information on the risks of death, disability, readmission, functional limitations, and access to services. Among these diseases, stroke stands out due to its high mortality and morbidity rates, with permanent sequelae that make post-discharge care a significant challenge, particularly due to the frequent need for hospital readmissions [5,6]. This challenge is especially relevant in Latin America, where countries face structural and access-related barriers to the longitudinal follow-up of stroke survivors [7].

The severity of impairments among stroke survivors may lead to unplanned hospital readmissions, often associated with discontinuity of home care, placing an added burden on health systems. The causes of readmission tend to recur, reflecting the clinical status of the survivor and requiring individualized post-discharge care interventions [8,9,10,11,12,13,14]. Socially vulnerable individuals who have suffered a stroke present a higher prevalence of risk factors such as low income, limited education, unemployment, and restricted access to healthcare, all of which hinder post-stroke recovery [15,16].

Social vulnerability is the process through which individuals or groups are exposed to conditions that increase their fragility and risk of exclusion, thus limiting access to fundamental rights such as health, education, housing, and employment. This concept goes beyond a static view by incorporating structural, historical, and contextual factors that intensify inequalities and limit opportunities [17,18].

This condition of vulnerability is especially pronounced in regions with low Human Development Index (HDI), reflecting inequalities in access to essential services such as healthcare, education, and income [19]. In Latin America, the demographic and socioeconomic characteristics of countries underscore the relevance of structural determinants in shaping health outcomes and the responsiveness of health systems [4,20].

The development of a predictive model based on ML that addresses social vulnerability may enable the identification of stroke survivors at high risk of readmission and pave the way for improved care management. The implementation of such a model aims to inform individualized interventions, guide public policy development, and highlight key elements, such as counter-referral services in primary healthcare (PHC), to which stroke survivors should be directed after their initial hospital discharge, following a coordinated care network logic.

From this perspective, the Chronic Conditions Care Model (CCCM), proposed by the World Health Organization (WHO) and structuring the Brazilian Care Networks for People with Chronic Diseases (RASPDC), seeks to improve population health by understanding individual and collective risks to which individuals are exposed, encouraging people and their families to engage in their own care. It incorporates health condition management technologies and case management, enabling preventive interventions on risk factors at various levels of the CCCM [21].

Understanding that the combination of clinical and social factors increases the likelihood of readmission reinforces the importance of ML-based predictive models that account for social vulnerability. Such models can identify stroke survivors at greater risk, guide individualized interventions, and support public policies aimed at restructuring care systems, particularly in contexts where structural inequalities still limit access to integrated and continuous health services. In this context, the present study aimed to develop a predictive model for hospital readmission among socially vulnerable stroke survivors.

## 2. Materials and Methods

The study employed a cross-sectional design [22], with data collection conducted from August to October 2023 and from April to May 2024, in primary healthcare (PHC) Units in two municipalities in northeastern Brazil characterized by low Human Development Index (HDI) and marked social inequality. The study report followed the Strengthening the Reporting of Observational Studies in Epidemiology (STROBE) guidelines [23].

The study population consisted of patients with a history of ischemic or hemorrhagic stroke, as documented in the follow-up records of the respective primary healthcare unit. Inclusion criteria were age 18 years or older, given the higher prevalence of stroke in adults [24]; and registration with the ESF in one of the municipalities. Exclusion criteria included acute diagnosis of Transient Ischemic Attack (TIA) or symptomatic cerebrovascular conditions reported by the patient, family member, or healthcare team; planned or scheduled readmissions; emergency care without hospitalization; follow-up or reassessment consultations; and death during hospitalization.

A simple random sample was calculated using G*Power^®^ software, version 3.1.9.7, with an a priori analysis of the required sample size for evaluating variables in contingency tables [25]. The effect size was set at 0.30, with an alpha error probability of 0.05 and power (1—beta error probability) of 0.80. The minimum required sample size with a 95% confidence interval was 143 participants. By the end of the study, 267 participants were included. Participants were randomly selected, and those who declined participation were replaced through the same random selection process.

The data collection instrument consisted of four groups of variables: socioeconomic, health condition, biological, and psychosocial variables. The dependent variable was hospital readmission, defined as hospital admission following the discharge date from the stroke-related hospitalization (first or only event). Both stroke-related and -unrelated readmissions were identified.

To assess functional dependence, the Barthel Index [26], was applied, classifying patients as: total dependence (≤25 points), severe dependence (26–50 points), moderate dependence (51–75 points), mild dependence (76–99 points), and fully independent (100 points) [26].

A pilot test was conducted with a population not included in the final sample to adjust the data collection instrument [27]. Data from the pilot were not used in the main study results. Participants were interviewed at home in a private setting to ensure confidentiality and anonymity, following prior scheduling, and accompanied by a Community Health Agent (ACS) or another team member.

Stroke can result in disabling complications, with up to 90.00% of patients experiencing expressive speech disorders [28], potentially preventing coherent responses to the questionnaire; in such cases, questions were directed to a family member or responsible caregiver. To minimize recall bias, documents issued by healthcare professionals/services, such as prescriptions, discharge reports, and test results, were reviewed.

Exploratory data analysis involved calculating descriptive statistics, such as frequencies, central tendency (mean), and dispersion (standard deviation). Subsequently, logistic regression and decision tree models were fitted to predict readmission. These models were selected based on the binary nature of the outcome variable (readmitted or not). To standardize variable scales and prevent variables with larger magnitudes from disproportionately influencing model performance, the Min–Max normalization technique was employed, transforming variable values to a 0–1 range.

For classification problems involving categorical variables, boundary plots were generated to identify optimal separation between classes, including for socioeconomic variables, health conditions, biological variables, functional dependence (assessed using the Barthel Index), and psychosocial variables.

Two predictive models were developed: logistic regression and decision tree, which were compared. The Ridge logistic regression model was adopted to minimize the risk of overfitting and multicollinearity among predictors. The model was optimized through stratified 5-fold cross-validation and GridSearchCV, testing different values of the regularization hyperparameter. Predictors were standardized using the StandardScaler method. In addition to the penalized version (Ridge), a classical unpenalized logistic regression model was also estimated to obtain the regression coefficients and odds ratios (OR), allowing for a direct comparison between both approaches. Confidence intervals were estimated using the bootstrap resampling technique with 1000 iterations.

The decision tree model applied was CART (Classification and Regression Tree), prioritizing clinical segmentation interpretability. The Gini index was used as the impurity criterion, and tree complexity was controlled using the ccp_alpha (Cost-Complexity Pruning) parameter. Multiple values of alpha were tested, and the one that maximized predictive performance on the validation set was selected. After this optimization, variables with relative importance lower than 2% were removed, and the model was recalibrated.

Both models were evaluated under three distinct validation schemes to test the robustness and stability of the results. Variable partitioning was performed in two groups—70% and 80% for training, and 30% and 20% for testing, respectively, for both model types—and through stratified 5-fold cross-validation (5KFold) to estimate mean performance and standard deviation across folds.

The performance metrics assessed included accuracy ≥0.70 (proportion of correct classifications), AUC-ROC > 0.75 (global discriminative ability), Cohen’s Kappa between 0.40 and 0.60 (agreement between observed and predicted values), RMSE < 0.50 (mean calibration error), as well as false positive and false negative rates.

All analyses were conducted using Python software (version 3.13), with the following libraries: pandas, numpy, matplotlib, scikit-learn, and seaborn. All analyses assumed a statistical significance level of 5% and were performed in Python.

This study followed all ethical and legal principles for research involving human subjects (Brazil, 2012; Brazil, 2016; Brazil, 2018). It was approved by the Research Ethics Committee (CEP) of the Federal University of Piauí (UFPI), under CAAE 67949523.4.0000.5214 and approval number 5.981.191. All participants signed the Informed Consent Form (ICF).

## 3. Results

### 3.1. Sample Characterization

Male participants comprised the majority of the sample (52.40%). The mean age was 70.5 years (SD = 12.1), with stroke cases occurring across a wide age range, from 38 to 111 years. Nearly all participants (88.40%) self-identified as Black or Brown. More than half (54.30%) lived with a partner. Most did not have a caregiver (58.40%), and among those who did, the majority were informal caregivers (37.80%).

Regarding health history and lifestyle, 75.30% had hypertension (HTN), 35.60% had diabetes mellitus (DM), 24.70% had experienced a previous stroke, 27.70% had dyslipidemia, 12.30% reported a cardiac comorbidity, 17.60% had pneumonia, and 17.20% had some type of dementia. Concerning primary health conditions presented by stroke survivors, 59.90% reported not seeking medical assistance prior to the index event. Among the strokes that occurred, 88.00% were ischemic strokes (IS).

In the overall sample, the prevalence of readmission at any point was 46.80%. Among these, 60.80% were readmitted for stroke-related causes. The prevalence of readmission within one year following the index event was 37.10%.

With regard to psychosocial interaction variables among stroke survivors, 83 participants (31.10%) reported some degree of difficulty sleeping after the index event. In addition, 16 (6.00%) and 82 (30.70%) presented with aphasia and dysphasia after the illness, respectively (Table 1).

### 3.2. Construction of ML Models

For the decision tree, the dataset was divided into two distinct parts: a training set (70%) used to train the ML model, and a test set (30%) used to evaluate the performance of the trained model on separate data. The model’s accuracy was 74.1%, demonstrating a better balance between false negative and false positive classifications. (Table 2).

The area under the ROC curve of the decision tree model for this partition was 80.3%. This value indicates that the model has a high capacity to correctly distinguish between patients who were readmitted and those who were not. Dividing the data into a training set (80%) and a test set (20%) allowed for evaluating the model’s generalization ability. The results indicate that, after training, the model performed better in classifying new patients (test set) with respect to the dependent variable ‘readmitted’.

Analysis of the confusion matrix showed that the model performed well in classifying patients. The accuracy was 70.4%, with 25.9% false positives, but only 3.7% false negative classification errors, characterizing high sensitivity but lower specificity (Table 2). For this partition, the area under the ROC curve of the decision tree model reached 81.8%, indicating an exceptional ability to correctly distinguish between readmitted and non-readmitted patients. This result demonstrates the model’s high precision in patient classification. The Decision Tree (CART) model evaluated through 5-fold cross-validation demonstrated consistent and reliable performance, with an average accuracy of 70.8% indicating balanced and consistent performance between false positive and false negative classifications. Furthermore, an AUC-ROC of 77.7% indicates acceptable discriminatory power and stability across validation folds.

Analysis of the confusion matrix for the logistic regression model with the 70%/30% partition revealed good performance in patient classification. The model had a 17.3% error rate by incorrectly classifying a patient who was not readmitted as readmitted (false positive) and a 16.0% error rate by misclassifying readmitted patients as not readmitted (false negatives). The accuracy based on available data was 66.7%, with a greater loss of sensitivity (Table 1). The area under the ROC curve of the logistic regression model reached 78.4%, indicating that the model has adequate capacity to correctly distinguish between patients who were readmitted and those who were not.

Analysis of the confusion matrix with the 80%/20% partition showed that the logistic model performed well in classifying patients. It correctly identified 27 patients who were not readmitted (true negatives) and 14 who were readmitted (true positives). However, the model made seven errors by incorrectly classifying patients who were not readmitted as readmitted (false positives) and six errors by misclassifying readmitted patients as not readmitted (false negatives), indicating balanced errors and good sensitivity. For this sample, the logistic classification model achieved an accuracy of 75.9% (Table 2).

For this partition (80% training and 20% testing), the area under the ROC curve of the logistic regression model reached 80.3%, showing a better balance between sensitivity and specificity. This result demonstrates the model’s good precision in patient classification. The Ridge Logistic Regression model assessed using 5-fold cross-validation achieved an average accuracy of 74.1% and an AUC-ROC of 80.4%, demonstrating good discrimination, moderate agreement, and stable performance across validation folds.

The models using a 70% training and 30% testing data split showed moderate performance, with good discrimination (AUC 78.4%) and moderate accuracy (66.7%). The Ridge Logistic Regression model with 5-fold cross-validation achieved a mean accuracy of 70.0% (±0.08) and a mean AUC-ROC of 77.9% (±0.084), indicating predictive stability and consistent performance across folds (Table 2).

Both models show moderate accuracies, with better performance observed for the Ridge Regression model (80/20) and the Decision Tree model (70/30). However, the cross-validation results demonstrate similar robustness, indicating model stability in the face of data variation. All models exhibited an AUC > 0.75, which is considered good discriminative performance, with the 80/20 Decision Tree model standing out. The Kappa values indicate moderate agreement between predictions, suggesting that both models adequately represented the readmission pattern. The root mean squared error (RMSE) values ranging from 0.41 to 0.48 indicate good calibration, with the Decision Tree model showing predicted probabilities closer to the observed outcomes.

**Figure 1:**
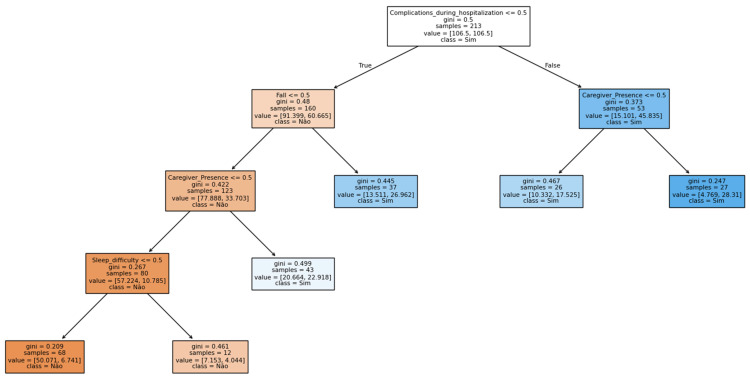
Decision tree of the most important explanatory variables for hospital readmission among stroke survivors. Data partitioning: 80% training/20% testing (stratified). Source: research data.

Among the most important explanatory variables for hospital readmission of stroke survivors in the decision tree model, using readmission within one year as the root variable, the splits selected complications during hospitalizations and falls as key health-related predictors; having a caregiver and difficulty sleeping were identified as the most important socioeconomic and psychosocial variables, respectively, in determining hospital readmission (Figure 1).

Both the decision tree and logistic regression models converge on key determinants of hospital readmission after stroke, particularly clinical complications during hospitalization and the presence of a caregiver, which highlight the dual importance of clinical stability and social support in post-stroke care. The integration of these findings within the Chronic Care for Conditions Model (CCCM) underscores the need for risk stratification, multidisciplinary follow-up, and continuity of care across levels of the health system. These results reinforce that combining predictive analytics with structured chronic care management can improve coordination, prevent avoidable readmissions, and enhance long-term outcomes for stroke survivors.

According to the logistic regression model, the explanatory variables (X_1_ … X_61_) with the greatest classification power are presented below. The variable with the highest coefficient is the most significant for the model’s classification—that is, for determining whether the patient will be readmitted or not. The eight most important variables for predicting readmission within one year are as follows. Acute clinical variables—complications during hospitalization, falls, skin lesions, and type of stroke—had the greatest weight in the risk of readmission (between 15% and 18%). Functional and social factors—caregiver presence, sleep difficulty, and time since the stroke in months—showed an intermediate influence (between 7% and 15%), while home care interventions demonstrated a mild protective effect (−3%) (Table 3).

Table 4 presents an interpretative framework of the results of the predictive models in light of the MACC.

## 4. Discussion

The prediction of hospital readmission among stroke survivors is essential for optimizing care and reducing recurrent hospitalizations. Predictive models based on the CCCM provide a robust theoretical framework that can enhance our understanding of the challenges faced in caring for this population. By aligning the principles of the CCCM with the results of this study, it becomes possible to more comprehensively assess the needs of post-stroke patients.

The analysis of the patients’ profiles indicates that factors such as advanced age, comorbidities, and unfavorable socioeconomic conditions increase vulnerability to hospitalization and clinical complications. Populations in situations of social vulnerability face significant barriers to accessing healthcare, which can worsen clinical conditions, increase frailty, and negatively affect functionality and quality of life. These findings highlight that the sociodemographic and clinical characteristics of patients directly influence the risk of hospital readmission, reinforcing the importance of care strategies tailored to these conditions. This perspective reinforces the concept of coordinated and continuous care, capable of anticipating demands, closing care gaps, and promoting a more structured approach to preventing readmissions.

The implementation of the predictive model in the investigated healthcare services can restructure the care provided to stroke survivors, fostering the development of routines and workflows that prioritize patients at higher risk of readmission. Identifying individuals at risk can lead to the creation of joint therapeutic plans involving primary healthcare teams, family members, and caregivers, thereby enhancing monitoring, enabling effective interventions, reducing the risk of readmission, and improving quality of life. The development of therapeutic plans that include preventive home measures represents promising strategies to be explored: the strengthening of self-care, symptom surveillance, and prompt health-seeking behavior, coordinated through community health workers.

The implementation of the CCCM and similar models derived from it in Latin America, promoted by the Pan American Health Organization (PAHO), emphasizes the fundamental importance of PHC and recognizes that the best clinical outcomes are achieved when all components of the model are interconnected and operate in a coordinated manner [29].

The analysis using the Decision Tree model, with a 70% training and 30% testing split, showed promising results in identifying predictive factors for readmission, with an accuracy of 74.1%, considered satisfactory for clinical application [30]. Models with accuracy above 75.00% are deemed relevant for clinical applications, provided they are accompanied by complementary analyses to minimize classification errors [31].

The AUC-ROC, a widely used metric in the evaluation of predictive models [32], was 80.3%, indicating good discriminative ability between readmitted and non-readmitted patients. This performance aligns with the reference threshold of an AUC-ROC above 85.00% as an indicator of reliable classification [33], and enables more robust analysis for clinical decision-making [34].

Tests using an 80/20 partition, common practice in model evaluation [4], demonstrated improved performance, with the decision tree showing greater predictive capacity due to the larger training dataset [35]. Despite this improvement, the model still presented limitations such as false positives and false negatives [33].

Logistic regression with a 70/30 partition achieved an AUC-ROC of 78.4% and accuracy of 66.7%, indicating adequate discriminative ability. Studies highlight that an AUC-ROC above 0.70 indicate acceptable discrimination, while values greater than 0.80 represent good predictive performance in clinical models [34]. However, the presence of false negatives is concerning in clinical settings, where at-risk patients may not be identified in time for preventive interventions [35]. The model showed the greatest loss of sensitivity compared to the decision tree model [36].

In the 80/20 partition, logistic regression achieved an AUC-ROC of 80.3% and an accuracy of 75.9%, demonstrating a performance gain with a larger training dataset [31]. Nonetheless, adjustments aimed at improving sensitivity and reducing false negatives are necessary to enhance clinical reliability [34]. The comparison between models revealed that in the 70/30 partition, decision tree slightly outperformed the logistic regression. However, in the 80/20 partition, the decision tree showed superior performance in both accuracy and AUC-ROC, standing out as an effective predictive tool to support clinical decision-making. However, the cross-validation (5-fold) results show similar robustness, indicating the stability of the models in the face of data variation.

Both models demonstrated satisfactory predictive performance, with accuracy and AUC-ROC values considered acceptable for clinical risk prediction models. These findings suggest that the decision tree model excels in interpretability and clinical applicability, being more sensitive for identifying patients at risk of readmission, whereas the Ridge regression model offers greater statistical robustness and lower overfitting tendency, supporting better generalizability.

This indicates that increasing the proportion of training data positively contributes to the performance of the decision tree model, highlighting the importance of fine-tuning ML models, such as parameter optimization and relevant variable selection, to achieve better predictive outcomes [35]. These findings suggest that both logistic regression and decision tree models have potential for use in predicting patient readmissions, each with distinct characteristics and strengths. The choice of the most appropriate model should be guided not only by performance metrics, but also by the specific needs of the clinical setting, as well as a consideration of the impacts that classification errors may have on patient care and management.

The analysis of hospital readmission among stroke survivors using a decision tree identified relevant predictive factors, such as complications during hospitalization and falls. Socioeconomic and psychosocial factors, such as the absence of a caregiver and difficulty sleeping, increase the likelihood of readmission, aligning with evidence that a lack of adequate support elevates the risk of complications [37]. This is a widely shared challenge among health systems in Latin America and the Caribbean, which are often marked by limited access to post-discharge care and home-based rehabilitation services [38].

In logistic regression, falls increased the likelihood of readmission corroborating Gaspari et al. (2019) [39]. Ischemic stroke raised the probability of readmission, reinforcing its severity [20,39]. Complications during hospitalization and skin lesions showed the strongest contribution to readmission risk, indicating that acute clinical instability and secondary physical complications significantly increase the likelihood of hospital return after stroke [13]. These results underscore the need for a comprehensive approach that considers clinical, social, and economic conditions, especially in contexts of high social vulnerability such as those observed in Latin American countries, where precarious social determinants of health amplify the risk of adverse events after hospital discharge [40].

Additionally, health systems in Latin America are mixed in nature, with joint participation of the public and private sectors. Each subsystem has its own financing model and adopts uncoordinated strategies in response to health issues, generating inequalities in the quality of care delivered. This fragmentation and segmentation result in a heavy burden of inequity, treating the human being as an object of the economy and disregarding their dignity. As a consequence, healthcare is often neglected, particularly for individuals with chronic conditions [41].

The structure of the CCCM (Microsystem, Mesosystem, and Macrosystem) provides a comprehensive framework for interpreting predictive models. At the microsystem level, aspects such as functional sequelae, home care, low adherence to therapies, and use of continuous medications can be observed. These factors highlight the urgency of strengthening the integration between primary care, specialized services, and rehabilitation programs, resonating with the need for coordinated healthcare networks, as advocated by recent public system reforms in Latin America aimed at overcoming fragmentation and promoting continuity of care [42]. Moreover, it is essential to expand family and community support to improve care for stroke survivors.

At the mesosystem level, the need to integrate rehabilitation services and psychosocial support is evident, especially for patients with high dependency and informal caregivers. Additionally, sleep difficulties and the lack of psychological support underscore the importance of expanded care networks. Health education and self-management remain challenges, particularly when working with vulnerable populations. Patients with low literacy levels and dependency on social benefits require adapted educational strategies. Furthermore, intersectoral coordination between health, social assistance, and education is essential to address inequalities and reduce readmissions. Socioeconomic vulnerability and the absence of formal support emphasize the need for intersectoral coordination, integrating health, social services, and other public policies to promote more favorable recovery and reduce disparities.

At the macrosystem level, structural determinants such as poverty, social exclusion, cultural values, racial and gender inequality, and low educational attainment limit the access to and continuity of care. These factors call for structural changes and public policies that promote a more equitable and inclusive social and health system. The CCCM reinforces the importance of public policies that integrate health and social determinants, promoting equity and better outcomes.

The study revealed weaknesses in the follow-up care of post-stroke patients, with many readmissions linked to complications that could have been prevented. Brazilian programs such as “Melhor em Casa” (“Better at Home”) have sought to address this demand by offering home care within the framework of the Unified Health System (SUS), but their coverage and effectiveness remain limited given the complexity of the cases [43]. The CCCM emerges as an alternative for more proactive management of chronic conditions, with the potential to better coordinate services and reduce hospital readmissions, provided that it is supported by structured public policies.

The decision tree presents five possible pathways that can be interpreted according to the levels of the Chronic Care for Conditions Model (CCCM). The first pathway represents patients without in-hospital complications or sequelae, who have caregivers and no sleep difficulties, resulting in a low risk of readmission. Care for this group could be managed at Level 3 of the CCCM, focusing on the management of chronic conditions. These individuals may receive routine follow-up, self-care promotion, mild rehabilitation, and annual monitoring.

The pathways representing “patients without acute complications or falls, but with sleep difficulties even when having a caregiver” and “patients without complications or falls, but without a caregiver” indicate an intermediate risk of readmission and can be placed at Level 4. These patients should have priority actions in primary healthcare (PHC) directed toward the implementation of individualized care plans, with expanded home visits and integration with the Family Health Support Center and multidisciplinary rehabilitation.

Finally, the pathways corresponding to “patients who experienced in-hospital complications and have a caregiver” and “patients with in-hospital complications and no caregiver” represent the most vulnerable profiles. Level 5 targets patients with a high probability of readmission—individuals whose pathways are marked by falls, time since stroke of less than 12 months, hemorrhagic stroke, or absence of a caregiver. At this level, given the severity, actions such as case management by PHC, intensive home care, specialized support, and coordination with hospital and rehabilitation services may be the most effective.

In other Latin American countries, similar initiatives face comparable challenges. In Costa Rica [44], the Long-Term Care program has emphasized home care, the training of formal caregivers, and family support, although budget constraints and legal uncertainties threaten its continuity. In Colombia [45], Home Care Programs for Chronic Patients aim to better integrate services but still face difficulties related to the diversity of clinical profiles and the organization of health teams’ work. These examples demonstrate that home care is a significant strategy for reorganizing health services; however, its effectiveness depends on investment, planning, and political will to function resolutely.

One limitation of the study was the difficulty patients and/or family members had in accurately recalling information about the disease. This recall bias was minimized by requesting exams, discharge reports, and medical prescriptions.

Another limitation of the study concerns the use of “hospital readmission” as a general outcome, encompassing both stroke-related and non-stroke-related causes, which may reduce the specificity of the identified predictors. However, this methodological choice was intentional and conceptually aligned with the notion that stroke survivors represent a population with chronic vulnerability. Following the acute event, patients experience functional, cognitive, and metabolic decline that predisposes them to a broad range of health complications and hospitalizations. Therefore, considering overall readmission captures the systemic and long-term impact of stroke and provides a clinically relevant indicator of post-stroke frailty. The use of a composite outcome was also justified by the sample size, as stratifying readmissions by cause would compromise statistical power. Future studies with larger cohorts may refine this model by distinguishing stroke-specific readmissions to increase predictive precision. Another limitation was that the sample consisted of patients from two municipalities in Northeastern Brazil with low Human Development Index (HDI), which restricts the generalizability of the model’s application. However, machine learning models, whenever transferred to settings other than those in which they were developed, must be tested and re-evaluated for accuracy prior to broader implementation.

Beyond these aspects already discussed, the study also adopted a methodological approach that reflects the complexity of post-stroke care. Within this broader context, the decision to adopt overall hospital readmission as the main outcome of the study was both strategic and theoretically grounded. Stroke is a marker of systemic vulnerability, and hospital readmissions reflect post-stroke frailty rather than merely neurological complications. Evidence indicates that a substantial proportion of readmissions occur due to non-neurological causes—such as infections, falls, cardiac complications, and metabolic disorders—demonstrating greater susceptibility to multiple health conditions and constituting a clinical marker of chronic vulnerability [9,10,13]. By adopting overall readmission as the outcome, the model enhances its usefulness for clinical management and primary healthcare, allowing for the stratification of complex patients’ risk, guiding personalized interventions, and supporting continuous surveillance within the logic of the Chronic Condition Care Model (CCCM). Thus, the model’s scope—focused on general readmission rather than stroke-specific recurrence—captures the multidimensional vulnerability of stroke survivors and provides a pragmatic tool for care management, aligned with the principles of the Chronic Care Model and the conceptual structure of the CCCM.

Among the study’s strengths were the high accuracy of the predictive models and the integration of multiple variables, considering not only clinical factors but also socioeconomic and psychosocial aspects. This provided a broader view of hospital readmissions among socially vulnerable patients. The use of the CCCM provided a solid theoretical framework, reinforcing the importance of intersectoral coordination in post-stroke care.

Furthermore, the proposed model can serve as a reference for the development of other machine learning-based predictive models, taking into account the framework of the Chronic Care Model (CCM), patients in situations of social vulnerability, and the outcome of hospital readmission among stroke survivors.

## 5. Conclusions

In a context where several Latin American countries have been striving to strengthen home care as part of their response to chronic conditions, this study developed predictive models of hospital readmission in stroke survivors living in situations of social vulnerability, based on the principles of the CCCM. The model demonstrated the ability to estimate the probability of readmission with adequate levels of sensitivity and specificity. The good accuracy rates and adequate area under the ROC curve indicate that the models have potential to assist in forecasting hospital readmissions and, consequently, in implementing more effective prevention strategies within post-stroke care.

The decision tree model was within the recommended standards for clinical risk models. Among the most relevant variables for predicting readmission were complications during hospitalization, occurrence of falls, presence of a caregiver, and difficulty sleeping. The analysis of socioeconomic characteristics, such as educational attainment and occupation, was also important, although the model proved more sensitive to clinical aspects such as stroke type and functional dependency.

Machine learning models can be incorporated into post-stroke patient follow-up protocols, prioritizing their use for risk stratification of hospital readmission and the planning of personalized care interventions. Integrating the model into electronic health record systems and developing simplified assessment tools based on the key predictive variables identified in this study may facilitate its routine use by healthcare teams. As a future perspective, multicenter studies are recommended to validate the model across different population contexts and to extend its application to other chronic conditions associated with readmission risk. The integration of such models into public healthcare policies for care management could enhance longitudinal surveillance of vulnerable patients and promote the continuous improvement of care quality.

## Figures and Tables

**Table 1:** Socioeconomic characteristics, health background, lifestyle, health conditions, and functional dependence of stroke survivors (n = 267). Teresina/Piauí, 2025.

Socioeconomic Characteristics		n (%)	Health Background and Lifestyle		n (%)	Health Conditions and Functional Dependence		n (%)
Sex	Male	140 (52.4)	Smoking (current or past)	Yes	99 (37.1)	Previous medical assistance before stroke	Yes	107 (40.1)
	Female	127 (47.6)		No	168 (62.9)		No	160 (59.9)
Age	(min/max)	(38/111) *	Alcohol use (current or past)	Yes	69 (25.8)	Type of stroke	Ischemic stroke	235 (88.0)
	Mean (SD)	70.5 (12.1)		No	198 (74.2)		Hemorrhagic stroke	20 (7.5)
Race/skin color	Non-white	241 (90.3)	Hypertension	Yes	201 (75.3)	Rehospitalized	Both	12 (4.5)
	White	26 (9.7)		No	66 (24.7)		Yes, stroke-related	76 (28.5)
Marital status	With partner	145 (54.3)	Diabetes Mellitus	Yes	95 (35.6)		Yes, other causes	49 (18.3)
	Without partner	122 (45.7)		No	172 (64.4)		Not rehospitalized	142 (53.2)
Education (years of schooling)	Illiterate	136 (50.9)	Previous stroke	Yes	66 (24.7)	Rehospitalized within one year after stroke	Yes	99 (37.1)
	Up to 8 years	102 (38.2)		No	201 (75.3)		No	168 (62.9)
	8 years or more	29 (10.9)	Dyslipidemia	Yes	74 (27.7)	Hospitalization location (first stroke)	Medical ward only	147 (55.1)
Income (minimum wage)	Less than one	17 (6.4)		No	193 (72.3)		Emergency room/ICU	57 (21.4)
	1 to <3	245 (91.8)	Dementia	Yes	46 (17.2)		Both (ward and emergency/ICU)	46 (17.2)
	3 to <5	5 (1.9)		No	221 (82.8)		Not hospitalized	17 (6.4)
Family composition	Min/max	1/9 *	Pneumonia	Yes	47 (17.6)	Better home care	Yes	143 (53.6)
	Mean (SD)	3.8 (1.6)		No	220 (82.4)		No	124 (46.6)
Place of residence	Urban area	219 (82.0)	COVID-19	Yes	58 (21.7)	Continuous medication use	Yes	249 (93.3)
	Rural area	48 (18.0)		No	209 (78.3)		No	18 (6.7)
Work activity	Yes	41 (15.4)	Falls	Yes	76 (28.5)	Functional dependence (Barthel Index)	Totally independent	102 (38.2)
	No	226 (84.6)		No	191 (71.5)		Slight dependence	54 (20.2)
Caregiver	No caregiver	156 (58.4)	Cardiac comorbidity	Yes	33 (12.3)		Moderate dependence	24 (9.0)
	Informal caregiver	101 (37.8)		No	234 (84.7)			
	Formal caregiver	10 (3.7)	Hospitalized complications	Yes	63 (23.6)		Severe dependence	23 (8.6)
				No	204 (76.4)		Total dependence	64 (24.0)

Source: Research data. Legend: * = minimum and maximum values; SD = standard deviation; Min = minimum; Max = maximum; ICU = Intensive Care Unit.

**Table 2:** Predictive Performance of Logistic Regression (Ridge) and Decision Tree (CART) Models for Hospital Readmission within One Year after Stroke.

Modelo	Validação	Acurácia	AUC-ROC	Kappa de Cohen	RMSE	Falsos Positivos n (%)	Falsos Negativos n (%)
Regressão Logística (Ridge)	80/20	75.9%	80.3%	0.48	0.48	7 (13.0%)	6 (11.1%)
	70/30	66.7%	78.4%	0.29	0.48	14 (17.3%)	13 (16.0%)
	5-KFold	70.0% ± 0.08	77.9% ± 0.084	0.36 ± 0.17	0.46 ± 0.01	15.75%	14.27%
Árvore de Decisão (CART)	80/20	70.4%	81.8%	0.43	0.41	14 (25.9%)	2 (3.7%)
	70/30	74.1%	80.3%	0.47	0.41	15 (18.5%)	6 (7.4%)
	5-KFold	69.6% ± 0.04	72.4% ± 0.04	0.36 ± 0.06	0.46 ± 0.01	17.6%	12.7%

Source: Research data.

**Table 3:** Explanatory most influential variables with the highest classification power for hospital readmission of stroke survivors according to the Classical Logistic Regression vs. Ridge Logistic Regression. Data partitioning 80% training/20% testing (stratified).

Variable	Classical Logistic Regression		Ridge Logistic Regression with Penalization for Control of Collinearity				Importance
	Coef Classic	Odds Ratio	Coef Ridge	Odds Ratio	95% Confidence Interval (Bootstrap with 1000 Resamples)		
					OR_2.5%	OR_97.5%	
Complications during hospitalization	0.4168	1.5171	0.0263	1.0267	1.0185	1.0463	18.17%
Fall	0.4761	1.6098	0.0264	1.0268	1.0155	1.0444	16.48%
Skin Lesion	0.4987	1.6466	0.0322	1.0327	1.0142	1.0428	16.21%
Type of Stroke	0.4172	1.5177	0.0054	1.0054	1.0125	1.0403	14.92%
Caregiver Presence	0.3849	1.4694	0.0287	1.0291	1.0133	1.0405	14.85%
Sleep difficulty	0.2151	1.24	0.0292	1.0296	1.0011	1.0295	8.78%
Time since stroke in months	−0.2019	0.8172	0.0156	1.0157	0.9768	0.9976	7.55%
Better at Home	0.1314	1.1404	−0.0134	0.9867	0.9918	1.02	3.04%

Source: research data.

**Table 4:** Interpretive framework of the results of the predictive models in light of the MACC.

CCCM Element	Finding from Decision Tree	Finding from Logistic Regression	Meaning in the Chronic Care Context	Level of Care/Professional Involved
Risk stratification and proactive care management	The variable “Complications during hospitalization” was the main decision node, indicating a higher risk of readmission.	The variable “Complications during hospitalization” showed the highest importance in predicting readmission risk.	Represents the need for early identification of complex cases and active follow-up by primary healthcare (PHC) after hospital discharge.	Primary and secondary care—multidisciplinary team (community health worker, physician, nurse, physiotherapist).
Supported self-care and patient engagement	The presence of a “Caregiver” strongly influenced the readmission outcome.	The presence of a “Caregiver” contributed to nearly 15% of the readmission outcome.	Reflects the role of family and community support in maintaining treatment adherence and preventing complications.	Primary care—nurse, community health worker.
Clinical decision support and multidisciplinary approach	Variables such as “Sleep difficulty” and “Fall” appear in the lower levels of the tree.	Fall, skin lesion, type of stroke, sleep difficulty, and time since stroke appear as predictors.	Demonstrates the need for an integrated clinical approach, considering functional and behavioral symptoms.	Primary care and rehabilitation (Family Health Support Centers)—physician, nurse, psychologist, physiotherapist.
Health information systems and continuous monitoring	The tree shows predictable readmission patterns based on simple clinical data.	The regression reveals predictable readmission variables based on simple clinical information.	Highlights the potential of health data use to guide interventions and continuous surveillance.	Health management and surveillance units—data analysts, program managers, coordination teams.
Integration across care levels and continuity of care	The model emphasizes the importance of communication between hospital and primary care after discharge.	Participation in the “Home Care Program” acted as a protective factor against readmission.	Indicates that lack of care coordination may contribute to avoidable readmissions.	Integrated Health Network (IHN)—care transition professionals, network managers.

Source: Research data.

## Data Availability

The data presented in this study are available upon request to the corresponding author.
